# Integrated Transcriptomics and Metabolomics Reveal Key Genes and Metabolic Pathway in Flower and Fruit Color Formation of *Cerasus humilis* (Bge.) Sok

**DOI:** 10.3390/plants14071103

**Published:** 2025-04-02

**Authors:** Shuai Zhang, Tianyuan Li, Shan Liu, Xinliang Qi, Yu Yang, Jiancheng Zhang, Luting Jia, Pengfei Wang, Xiaopeng Mu

**Affiliations:** College of Horticulture, Shanxi Agricultural University, Jinzhong 030801, China; szhang@sxau.edu.cn (S.Z.); 17735426816@163.com (T.L.); 18536495802@163.com (S.L.); 15263450921@163.com (X.Q.); 15513999787@163.com (Y.Y.); zjcnd001@163.com (J.Z.); jialuting@sxau.edu.cn (L.J.); pengfeiwang2004@163.com (P.W.)

**Keywords:** *Cerasus humilis*, flower and fruit color difference, anthocyanins, metabolome, transcriptome

## Abstract

Anthocyanins play a pivotal role in determining the color diversity in the flowers and fruits of *Cerasus humilis* (Bge.) Sok. This study performed a metabolomic analysis of the flowers and fruits of two varieties differing in pigmentation phenotypes (‘Jinou 1’ and ‘Nongda 5’), and the results indicated that the cyanidin, pelargonidin, paeonidin, and delphinidin were the main substances serving as the primary pigments contributing to their striking chromatic divergence between two varieties. Transcriptome profiling revealed that several key structural genes (*ChCHS1*, *ChDFR*, *ChF3H*, and *ChF3’H*) in the anthocyanin biosynthesis pathway exhibited significantly elevated expression levels in ’Jinou 1’ compared to ’Nongda 5’. Further metabolomic and transcriptomic correlation analyses identified that *ChMYB9* and *ChMYB12* exhibited strong positive associations with anthocyanin pathway metabolites in both floral and fruit tissues. Notably, *ChMYB9* displayed the strongest correlation with the metabolite profiles, suggesting it may serve as a core regulatory component of the anthocyanin biosynthesis. This research provides new insights into the regulatory mechanisms of anthocyanin biosynthesis in *C. humilis*.

## 1. Introduction

*Cerasus humilis* (Bge.) Sok., commercially known as “Calcium Fruit”, belongs to the subgenus *Prunus* of the Rosaceae family. It is characterized by easy flowering and early fruiting, and the fruit of *C. humilis* is rich in organic acids, calcium, vitamin C, and flavonoids [1]. The *C. humilis* germplasm exhibits remarkable color polymorphism in flowers and fruits. Varieties with pink flowers have higher ornamental value, while varieties with yellow fruits have better fresh-eating quality [2]. Therefore, to meet market demand, rapid screening of *C. humilis* varieties, particularly those with pink flowers and yellow fruits, is one of the most urgent breeding objectives.

Anthocyanins are secondary metabolites in plants classified within the flavonoid subclass of polyphenolic compounds, imparting rich colors to flowers, fruits, and leaves [3]. The biosynthetic pathway of anthocyanins is relatively conserved among plants and has been extensively studied in model plants [4]. The synthesis process can be divided into three stages, with key enzymes and genes well characterized throughout these stages [5,6]. The first stage involves the conversion of phenylalanine into 4-coumaroyl-CoA catalyzed by *PAL*, *C4H,* and *4CL*. The second stage is the transformation of 4-coumaroyl-CoA into flavanones via *CHS*, *CHI*, and *F3H*. In the third stage, flavanones are converted into unstable anthocyanins by the action of *DFR* and *ANS*, while stable anthocyanin products are formed through modifications by *UFGT* [7].

The structural genes of the anthocyanin biosynthetic pathway primarily encode enzymes involved in the flavonoid synthetic process, such as *PAL*, *CHS*, *CHI*, *F3H*, *F3’H*, *F3’5’H*, *DFR*, *ANS*, and *UFGT* [8]. Studies have shown that the expression of *CHS* and *CHI* can enhance anthocyanin accumulation in *Anthurium andraeanum* and *Muscari armeniacum* [9,10]. Inhibition of the activities of *MdF3H*, *MdDFR*, *MdANS*, and *MdUFGT* can reduce anthocyanin content in apple fruits [11,12]. The activity of key enzyme genes in the anthocyanin biosynthetic process is transcriptionally regulated by interactions between transcription factors and their downstream structural genes [13]. Typically, *MYB*, *bHLH*, and *WD* transcription factors form a ternary complex (MBW) for transcriptional regulation [12]. Among these, *MYB* is the most crucial transcription factor in regulating anthocyanin synthesis, directly influencing its production [14]. For instance, the R2R3-MYB transcription factors *PpMYB10.2* and *PpMYB9* in peaches can activate the transcription of anthocyanin biosynthetic genes [15]. *PyMYB10* and *PyMYB114* regulate anthocyanin biosynthesis in pear fruits by forming MBW transcription complexes [16]. In studies on the color regulation mechanisms of roses, two MBW complexes (*RcMYB1-RcBHLH42-RcTTG1*; *RcMYB1-RcEGL1-RcTTG1*) were found to control anthocyanin accumulation [17]. Considering the phylogenetic position of *C. humilis* within Rosaceae, we hypothesize that the variations in floral and fruit pigmentation of *C. humilis* are determined by anthocyanin biosynthetic genes, whose transcription may be governed by a conserved MYB-bHLH-WD40 regulatory complex.

In earlier studies, our research team measured anthocyanin content in fruits of different colored varieties of *C. humilis*, finding that red fruited varieties contained higher anthocyanin levels in their fruits than yellow fruited varieties [18]. Analysis of structural genes in the anthocyanin pathway revealed that *ChCHS*, *ChCHI*, *ChF3H*, *ChDFR*, *ChANS*, and *ChUFGT* had significantly higher expression levels in red fruited varieties of *C. humilis* [19]. Therefore, variety ‘Jinou 1’ (red flowers, red fruits) and variety ‘Nongda 5’ (white flowers, yellow fruits) were selected as research materials to investigate variations in anthocyanin biosynthesis of *C. humilis*. Metabolomic and transcriptomic sequencing results indicated that the main substances responsible for the color differences in flowers and fruits of the two varieties were cyanidin, pelargonidin, paeonidin, and delphinidin, while *ChCHS1*, *ChDFR*, *ChF3H*, and *ChF3’H* were the key structural genes associated with the observed differences. Further metabolomic and transcriptomic analyses identified that *ChMYB9*, a transcription factor encoded gene, correlates significantly with the anthocyanin pathway metabolites in flowers and fruits of *C. humilis*. This study provides a basis for exploring key genes that regulate the coloring mechanisms of *C. humilis* flowers and fruits, and for elucidating the coloration mechanisms related to flower and fruit traits in *C. humilis*.

## 2. Results

### 2.1. Phenotypic Differences in Flowers and Fruits of ‘Jinou 1’ and ‘Nongda 5’

**Figure 1:**
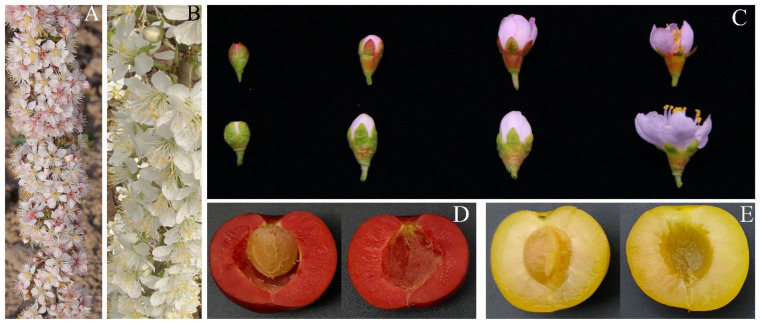
Phenotypes of ‘Jinou 1’ and ‘Nongda 5’. (**A**) ‘Jinou 1’ flowering period. (**B**) ‘Nongda 5’ flowering period. (**C**) Flower development in ‘Jinou 1’ (up) and ‘Nongda 5’(down). (**D**) Mature fruit of ‘Jinou 1’. (**E**) Mature fruit of ‘Nongda 5’.

Observations were made on the flowers of ‘Jinou 1’ and ‘Nongda 5’ at different developmental stages, and it was found that ‘Jinou 1’ exhibited a stable pink pigmentation in both sepals and petals from the earliest bud stages, with this hue persisting through petal expansion to full anthesis, whereas the sepals and petals of ‘Nongda 5’ remained white across all developmental phases (Figure 1A–C). Colorimetric analysis further revealed that the values of lightness (L*), red-green chromaticity (a*), yellow-blue chromaticity (b*), chroma (C*), and hue (H*) of ‘Jinou 1’ were 77.54, 2.41, 4.65, 5.24, and 62.60, respectively, while those for ‘Nongda 5’ were 79.91, 0.86, 5.25, 5.32, and 80.70, respectively.

Moreover, chromatometric measurements revealed that mature fruit of ‘Jinou 1’ exhibited a deep red phenotype (Figure 1D), characterized by low lightness (L* = 32.84) and exceptionally high red chromaticity (a* = 26.02), with moderate yellow contribution (b* = 13.03). This combination produced a vivid color intensity (C* = 26.60) and distinct red hue angle (H* = 29.10). Conversely, mature fruit of ‘Nongda 5’ displayed a bright golden-yellow coloration (Figure 1E), demonstrating significantly higher brightness (L* = 51.23) and dominant yellow chromaticity (b* = 36.26), accompanied by minimal red pigmentation (a* = 9.17). Notably, ‘Nongda 5’ achieved remarkable color purity (C* = 75.81) with a hue angle (H* = 37.40) positioned in the yellow-green spectral range.

### 2.2. Metabolomic Analysis of Flowers and Fruits of ‘Jinou 1’ and ‘Nongda 5’

Metabolomic profiling was conducted to elucidate the anthocyanin profiles in floral tissues of ‘Jinou 1’ and ‘Nongda 5’. Based on the KEGG compound database coupled with multiple reaction monitoring (MRM)-based quantification, we identified a total of 49 color-associated metabolites, including eight cyanidin derivatives, eleven delphinidin derivatives, two malvidin derivatives, five pelargonidin derivatives, three paeonidin derivatives, seven petunidin derivatives, five proanthocyanidin derivatives and eight flavonoid derivatives. Comparative analysis revealed remarkable metabolic diversity between the two cultivars: ‘Jinou 1’ contained 47 detectable anthocyanins, while ‘Nongda 5’ presented 43. Notably, six anthocyanins were uniquely identified in ‘Jinou 1’ flowers, including cyanidin-3-*O*-rutinoside, cyanidin-3-*O*-glucoside, cyanidin-3-*O*-arabinoside, cyanidin-3,5-*O*-diglucoside, delphinidin-3-*O*-glucoside, and pelargonidin-3-*O*-glucoside. Conversely, delphinidin-3,5-*O*-diglucoside and paeonidin-3-(6-*O*-p-coumaroyl)-glucoside were exclusively detected in ‘Nongda 5’ (Supplementary Table S1).

**Figure 2:**
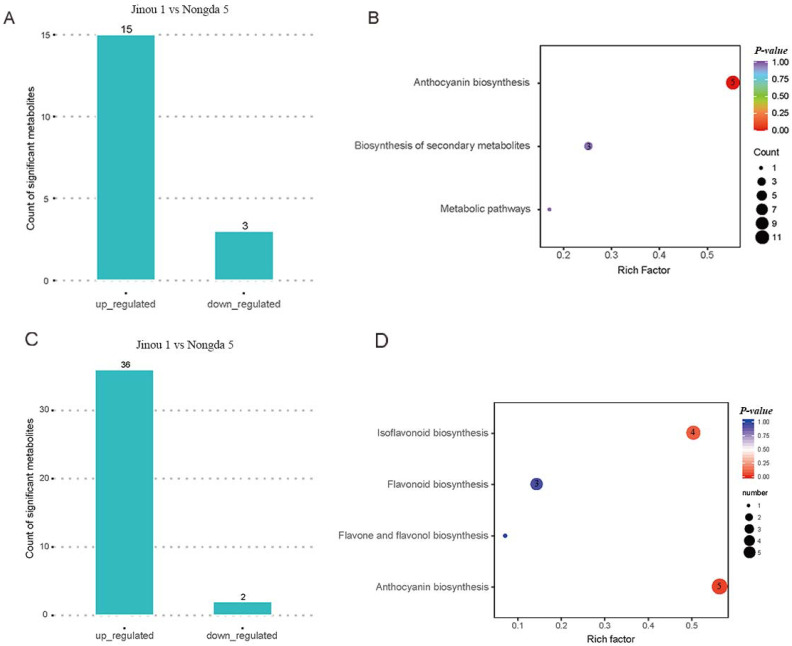
Analysis of metabolomic data of *C. humilis* flowers and fruits. (**A**) Numbers of differential metabolites in ‘Jinou 1’ vs. ‘Nongda 5’ flowers. (**B**) KEGG pathway enrichment analysis of differential metabolites in ‘Jinou 1’ vs. ‘Nongda 5’ flowers. (**C**) Numbers of differential metabolites in ‘Jinou 1’ vs. ‘Nongda 5’ fruits. (**D**) KEGG pathway enrichment analysis of differential metabolites in ‘Jinou 1’ vs. ‘Nongda 5’ fruits.

Applying rigorous selection criteria of fold change ≥2 and fold change ≤0.5, a total of 18 differential metabolites were identified, distinguishing ‘Jinou 1’ and ‘Nongda 5’. ‘Jinou 1’ displayed significantly elevated levels of 15 metabolites compared to ‘Nongda 5’, while three metabolites exhibited marked reduction (Figure 2A). Subsequent KEGG-based functional annotation of the 18 differential metabolites indicated that 11 metabolites were enriched in anthocyanin biosynthesis, three metabolites were mapped to the biosynthesis of secondary metabolites, and one metabolite was annotated in metabolic pathways. Notably, hierarchical clustering of pathway-specific metabolites highlighted profound compositional differences in anthocyanin biosynthesis between the two cultivars (Figure 2B).

Flavonoid profiles in the fruits of ‘Jinou 1’ and ‘Nongda 5’ were also characterized using metabolomic techniques, employing the same analytical pipeline as that in the floral metabolome study, this approach identified a total of 38 metabolites with significant differential abundance (≥2-fold change) (Figure 2C). KEGG functional annotation revealed that 12 metabolites were classified in the anthocyanin pathway, with five metabolites in anthocyanin biosynthesis, four metabolites in isoflavonoid biosynthesis, three metabolites in flavonoid biosynthesis, and one metabolite in flavone and flavonol biosynthesis (Figure 2D). Notably, eight anthocyanin-related metabolites including peonidin-*O*-hexoside, pelargonidin-3-*O*-malonylhexoside, cyanidin-3-*O*-glucoside, cyanidin-3,5-*O*-diglucoside, pelargonin, pelargonidin-3-*O*-beta-D-glucoside, peonidin-3-*O*-glucoside chloride, and malvidin-3-acetyl-5-diglucoside were uniquely detected in ‘Jinou 1’, while cyanidin-3-*O*-galactoside, cyanidin-*O*-syringic acid, cyanidin-3-*O*-rutinoside, and delphinidin-3-*O*-glucoside exclusively accumulated in ‘Nongda 5’ (Supplementary Table S2).

Principal component analysis (PCA) was conducted on the metabolomic datasets derived from flowers and fruits of ‘Jinou 1’ and ‘Nongda 5’ (Supplementary Figure S1). The PCA score plots revealed distinct clustering patterns between the two cultivars across both tissue types, with clear segregation along the primary principal components (PC1/PC2). This ordination pattern indicates profound metabolomic differences in floral and fruit metabolites between ‘Jinou 1’ and ‘Nongda 5’.

### 2.3. Transcriptomic Analysis of Flowers and Fruits of ‘Jinou 1’ and ‘Nongda 5’

**Figure 3:**
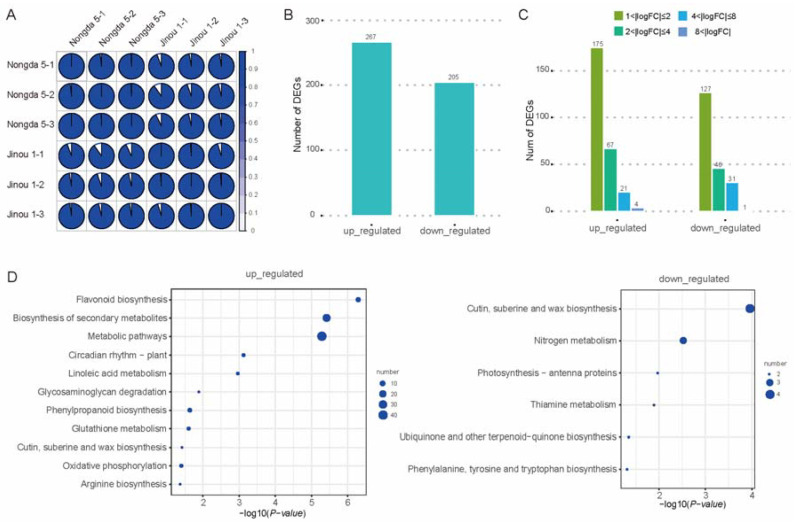
Analysis of transcriptomic data of *C. humilis* flowers. (**A**) Correlation analysis between ‘Nongda 5’ and ‘Jinou 1’ flower transcriptomic data. (**B**) Numbers of DEGs in ‘Jinou 1’ vs. ‘Nongda 5’ flowers. (**C**) Distribution of FC values of DEGs. (**D**) KEGG pathway enrichment analysis of DEGs for ‘Jinou 1’ vs. ‘Nongda 5’ flowers.

High-quality RNA extracted from fully bloomed petals of ‘Jinou 1’ and ‘Nongda 5’ was utilized to construct strand-specific cDNA libraries. Sequencing was performed on the NovaSeq 6000 platform, at Beijing Biomarker Bioinformatics Technology Co., Ltd. (Beijing, China), using paired-end 150 bp mode after rigorous quality control filtering (Figure 3A). Each biological replicate generated 20.7–27.9 million clean reads with Q30 scores exceeding 93.8%, ensuring robust data reliability. De novo transcriptome assembly using Trinity and all full-length transcripts were functionally annotated through homology searches against public databases. Differentially expressed genes (DEGs) were analyzed using the criteria |log2FC| > 1 and FDR < 0.05, and the results indicated that a total of 472 DEGs were identified between the two varieties. Specifically, 267 genes exhibited upregulated expression in ‘Jinou 1’ petals relative to ‘Nongda 5’, while 205 genes showed downregulation (Figure 3B,C). DEG enrichment analyses with standards *p* < 0.05 and *p* < 0.01 revealed that 118 genes were upregulated in ‘Jinou 1’, primarily distributed in metabolic pathways, biosynthesis of secondary metabolites, and flavonoid biosynthesis (Figure 3D).

**Figure 4:**
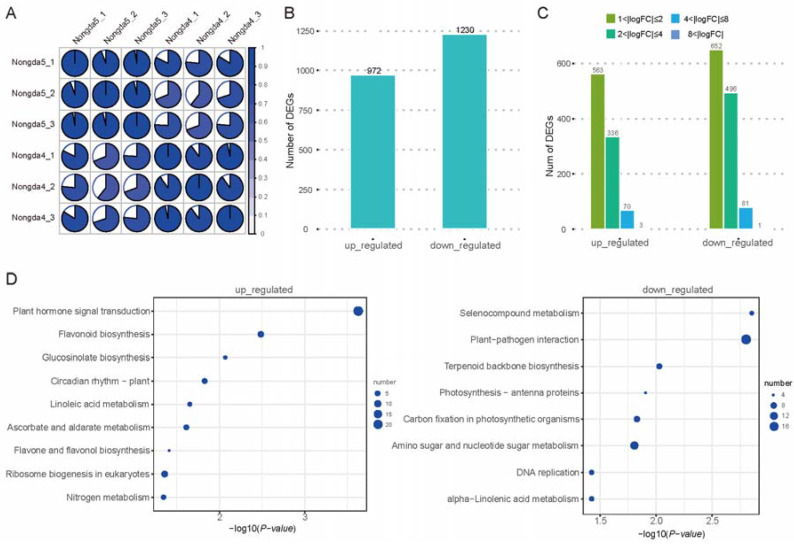
Analysis of transcriptomic data of *C. humilis* fruits. (**A**) Correlation analysis between ‘Nongda 5’ and ‘Jinou 1’ fruit transcriptomic data. (**B**) Numbers of DEGs in ‘Jinou 1’ vs. ‘Nongda 5’ fruits. (**C**) Distribution of FC values of DEGs. (**D**) KEGG pathway enrichment analysis of DEGs for ‘Jinou 1’ vs. ‘Nongda 5’ fruits.

Mature fruits from ‘Jinou 1’ and ‘Nongda 5’ were subjected to high-throughput RNA sequencing, yielding a total of 40.55 Gb of high-quality data, with each sample achieving 6.08 Gb of clean data (Q30 > 93%) for further analysis (Figure 4A). Employing the same Trinity-based *de novo* assembly and differential expression analysis pipeline, we identified a total of 2202 DEGs between the two varieties. Compared to ‘Nongda 5’, ‘Jinou 1’ had 972 upregulated genes and 1230 downregulated genes (Figure 4B,C). KEGG analysis showed that 57 genes were upregulated in ‘Jinou 1’, primarily distributed in plant hormone signal transduction, ribosome biogenesis in eukaryotes, and flavonoid biosynthesis (Figure 4D).

### 2.4. Analysis of Anthocyanin Pathway Genes in Flowers and Fruits

**Figure 5:**
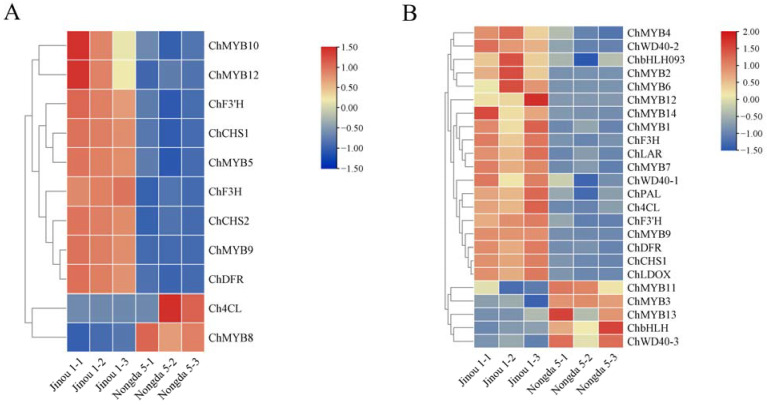
Heatmap of DEGs in *C. humilis* flowers (**A**) and fruits (**B**).

Comparative transcriptomic analysis of anthocyanin biosynthesis pathways revealed genotype-specific expression patterns between ‘Jinou 1’ and ‘Nongda 5’. In floral tissues, six key structural genes (*ChCHS1*, *ChCHS2*, *ChDFR*, *ChF3H*, *ChF3’H*, and *ChLAR*) showed significantly higher expression in ‘Jinou 1’ compared to ‘Nongda 5’, while *Ch4CL* exhibited lower expression levels in ‘Jinou 1’ (Figure 5A). Fruit transcriptomes displayed more pronounced differential regulation, with all eight annotated anthocyanin pathway genes (*ChPAL*, *Ch4CL*, *ChCHS1*, *ChDFR*, *ChF3H*, *ChF3’H*, *ChLDOX*, *ChLAR*) showing significantly elevated expression in ‘Jinou 1’. Notably, *Ch4CL* was upregulated in ‘Jinou 1’ fruits, suggesting tissue-specific regulatory mechanisms (Figure 5A).

Comparative transcriptomic profiling also identified 19 transcription factors (TFs) associated with anthocyanin regulation, including 14 MYB, 3 WD40, and 2 bHLH family members. In the flower petals, five MYB encoding genes were annotated, with *ChMYB5*, *ChMYB9*, *ChMYB10*, and *ChMYB12* showing significant upregulation in ‘Jinou 1’, while *ChMYB8* displayed lower expression in ‘Jinou 1’. In the mature fruits, seven *MYB* genes, two *WD* genes, and one *bHLH* gene showed higher expression in ‘Jinou 1’, while three *MYB* genes, one *WD* gene, and one *bHLH* gene were upregulated in ‘Nongda 5’ (Figure 5B).

### 2.5. Correlation Analysis of Transcriptome and Metabolome of ‘Jinou 1’ and ‘Nongda 5’ Flowers and Fruits

**Figure 6:**
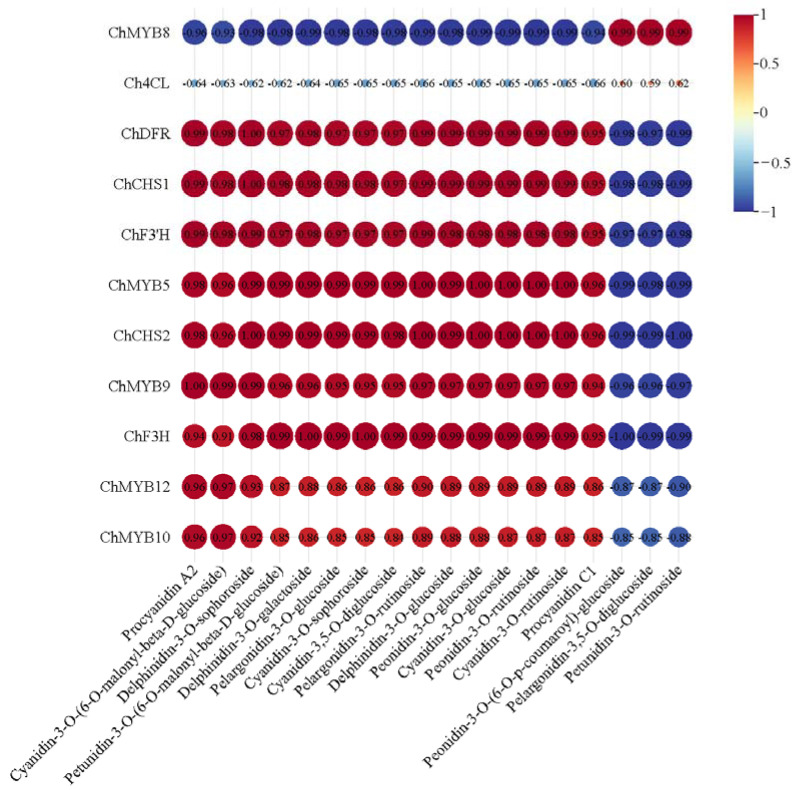
Correlation analysis between DEGs and differential metabolites in ‘Jinou 1’ vs. ‘Nongda 5’ flowers.

Integrated transcriptomic and metabolomic profiling of floral tissues from ‘Jinou 1’ and ‘Nongda 5’ revealed that three anthocyanin metabolites (peonidin-3-*O*-(6-*O*-p-coumaroyl)-glucoside, pelargonidin-3,5-*O*-diglucoside, and petunidin-3-*O*-rutinoside) were positively correlated with *Ch4CL*, but showing lower correlation with *ChCHS1*, *ChCHS2*, *ChDFR*, *ChF3H*, and *ChF3’H*. Additionally, these metabolites exhibited strong positive correlation with the MYB transcription factor encoding gene *ChMYB8*, yet displayed negative correlations with *ChMYB5*, *ChMYB9*, *ChMYB10*, and *ChMYB12*. The correlation relationships of the remaining 15 metabolites with structural genes of the anthocyanin pathway and *MYB* genes were the opposite (Figure 6).

**Figure 7:**
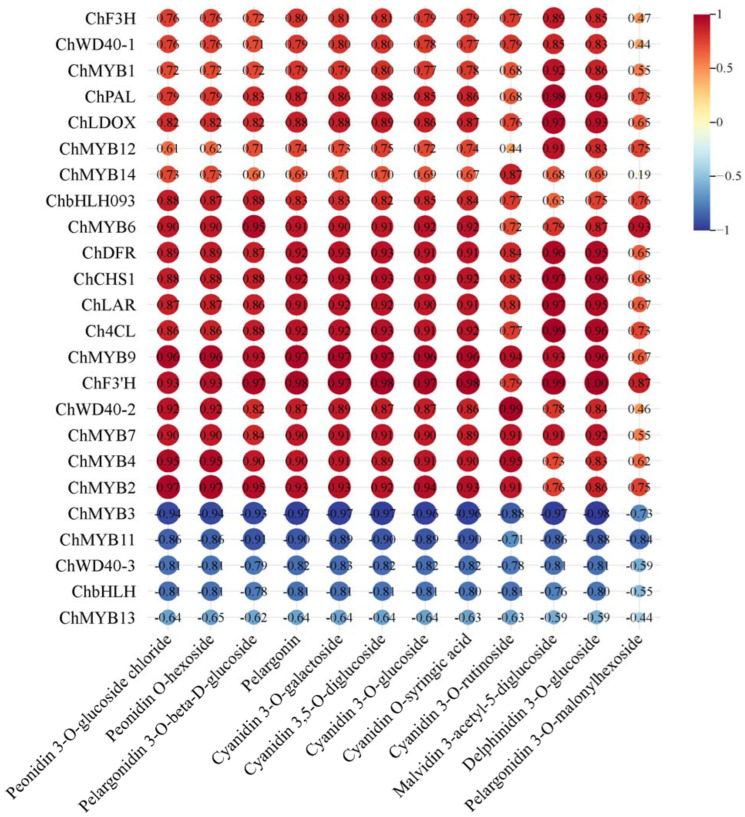
Correlation analysis between DEGs and differential metabolites in ‘Jinou 1’ vs. ‘Nongda 5’ fruits.

The combined analysis of the fruit transcriptome and metabolome from ‘Jinou 1’ and ‘Nongda 5’ indicated that among 12 detected anthocyanin metabolites, 11 metabolites showed significant positive correlations with eight structural genes (*ChPAL*, *Ch4CL*, *ChCHS1*, *ChDFR*, *ChF3H*, *ChF3’H*, *ChLDOX*, *ChLAR*). The exception was pelargonidin-3-O-malonylhexoside, which showed only weak positive association with these genes. Correlation network analysis between transcription factors and metabolites further elucidated that *ChMYB3*, *ChMYB11*, *ChMYB13*, *ChWD40-3*, and *ChbHLH* were negatively correlated with 12 metabolites, while the remaining 10 transcription factor encoding genes were positively correlated with the metabolites (Figure 7).

## 3. Discussion

Floral and fruit pigmentation represent critical traits that significantly influence ornamental and commercial value in horticultural plants [20,21]. In our previous study, the L* value and b* value of ‘Nongda 5’ were higher than those of ‘Jinou 1’, and the a* value of ‘Jinou 1’ was higher than that of ‘Nongda 5’ [18]. In this study, the color parameter differences observed in flowers and fruits of ‘Jinou 1’ and ‘Nongda 5’ indicated that the contrasting pigmentation phenotypes of *C. humilis* varieties ‘Jinou 1’ (pink flowers, red fruits) and ‘Nongda 5’ (white flowers, yellow fruits) provide an exceptional model system for dissecting color formation mechanisms.

Anthocyanins are widely distributed water-soluble pigments in plants, imparting various colors to plant organs and tissues [22]. Light-induced anthocyanin biosynthesis experiments demonstrated enhanced pigment accumulation correlating with intensified petal coloration in *Lilium* spp. and *Chrysanthemum morifolium* [23,24]. In *Paeonia suffruticosa* Andr., cyanidin-3,5-di-*O*-glucoside (Pn3G5G) was identified as the key pigment responsible for the formation of characteristic floral spots [25]. Similarly, *Rhododendron rex* petal pigmentation was attributed to a combination of four anthocyanins: pelargonidin, cyanidin-3,5-*O*-diglucoside, cyanidin-3-*O*-glucoside, and delphinidin [26]. Comparative metabolomics across *Lagerstroemia indica* cultivars revealed cyanidin, delphinidin, petunidin, and malvidin as the primary differentiators of flower color diversity [27]. This study performed targeted sequencing of the anthocyanin metabolome in the petals of two *C. humilis* varieties, ‘Jinou 1’ and ‘Nongda 5’, identifying 49 distinct anthocyanin metabolites, with eight differentially accumulated metabolites (Supplementary Figure S2). Among these, six uniquely accumulated metabolites in ‘Jinou 1’ belonged to cyanidin, delphinidin, and pelargonidin subclasses. Further analysis indicated that cyanidin-3-*O*-glucoside exhibited the highest concentration differential, suggesting that this compound is a major contributor to the flower color difference between ‘Jinou 1’ and ‘Nongda 5’.

In studies of substances responsible for fruit color differences, cyanidin has been identified as the primary anthocyanin modulating chromatic diversity across fruit species [28,29,30]. In this study, 12 different anthocyanin metabolites were detected in the petals of ‘Jinou 1’ and ‘Nongda 5’, including five cyanidins, three pelargonidins, two paeonidins, one malvidin, and one delphinidin. These could be the primary substances responsible for the distinct fruit color phenotypes of ‘Jinou 1’ and ‘Nongda 5’ (Supplementary Figure S3). Notably, cyanidin-3-*O*-glucoside emerged as the sole anthocyanin uniquely expressed in ‘Jinou 1’ fruits and differentially accumulated in petals between the two cultivars. Thus, we speculate that cyanidin-3-*O*-glucoside is a major contributor to the color differences in both the petals and fruits of ‘Jinou 1’ and ‘Nongda 5’.

The structural genes governing anthocyanin biosynthesis have been extensively characterized across plant species [31]. These genes are hierarchically organized into two functional modules based on their catalytic roles in the metabolic cascade: early biosynthetic genes (EBGs) encompassing *CHS*, *CHI*, *F3H*, and *F3’H*, and late biosynthetic genes (LBG) including *F3’5’H*, *DFR*, *ANS*, and *UFGT* [32]. EBGs are typically located upstream in the anthocyanin synthesis pathway; manipulating these genes can alter the flower or fruit color of plants. For instance, silencing the *CHS* gene in petunias can change the flower color from purple to white, while reducing *CHS* activity in tomatoes can lighten the fruit color [33,34]. LBGs, operating downstream, catalyze the formation of colored anthocyanins from their colorless precursors. For example, *DFR* is essential for producing leucoanthocyanidins (colorless delphinidin, pelargonidin, and cyanidin), and mutations in the *ANS* gene can block the conversion of leucoanthocyanidin to colored pigments [35,36,37]. Previous studies have found that the expression levels of *ChCHS*, *ChF3H*, and *ChDFR* genes were positively correlated with anthocyanin content, and the expression levels in ‘Jinou 1’ fruits were higher than those in ‘Nongda 5’ fruits [18]. In this study, the expression levels of *ChCHS1*, *ChDFR*, *ChF3H*, and *ChF3’H* were higher in the petals and fruits of ‘Jinou 1’ compared to ‘Nongda 5’. The combined results of transcriptome and metabolome analyses indicated that these four structural genes were positively correlated with anthocyanin metabolites, suggesting their significant roles in the accumulation pathway of anthocyanins in *C. humilis* flowers and fruits. Notably, tissue-specific expression divergence was also observed for other pathway genes. *ChPAL*, *ChLDOX*, and *ChLAR* exhibited differential expression patterns exclusively in fruits, while *Ch4CL* displayed reciprocal expression trends between flowers and fruits of the two varieties. These findings suggest complex regulatory networks underpin variety-specific anthocyanin profiles, with distinct gene sets controlling pigmentation in floral versus fruit tissues.

MYB, bHLH, and WD transcription factors promote or inhibit the biosynthesis of anthocyanins by recognizing and binding to specific promoter regions of structural genes. *MYB11*, *MYB12*, *MYB111*, and *MYB75/PAP1* can regulate the expression of EBGs [38]. Recent studies have shown that MYB, bHLH, and WD can form MBW complexes that enhance or suppress the expression of structural genes involved in the anthocyanin biosynthetic pathway [39]. The R2R3-MYB forms a complex with bHLH and WD to promote the expression of *DFR*, *ANS*, and *UFGT*, leading to the accumulation of anthocyanins in plants [40,41]. In this study, 10 *MYB* genes, two *WD* genes, and one *bHLH* gene were positively correlated with anthocyanin pathway metabolites, suggesting that these genes may play significant roles in the formation of anthocyanins in flowers and fruits of *C. humilis*. *ChMYB3* was negatively correlated with flower anthocyanin metabolites, while *ChMYB8*, *ChMYB11*, *ChMYB13*, *ChWD40-3*, and *ChbHLH* were negatively correlated with fruit anthocyanin metabolites, implying combinatorial control by multiple transcription factors across tissues.. The common *MYB* genes found in both flowers and fruits were *ChMYB9* and *ChMYB12*, both of which were positively correlated with anthocyanin metabolites. Notably, *ChMYB9* showed a highly significant positive correlation with anthocyanin metabolites. Therefore, we hypothesize that *ChMYB9* may be a key MYB encoding gene in the anthocyanin biosynthetic pathway of *C. humilis* flowers and fruits.

While this study offers novel insights into the transcriptional regulation of anthocyanin biosynthesis in *C. humilis*, several limitations warrant consideration. Notably, the candidate genes associated with floral and fruit coloration were identified through correlation analyses, yet no functional validation of these genes was performed. To address these gaps, future research should prioritize functional characterization of *ChMYB9*. Specifically, homologous or heterologous overexpression and knockdown studies in model systems or *C. humilis* itself could verify whether *ChMYB9* acts as a pleiotropic regulator of both floral and fruit coloration.

## 4. Materials and Methods

### 4.1. Materials

The *C. humilis* materials used in this experiment were collected from the *C. humilis* germplasm repository at Shanxi Agricultural University, located in Taigu District, Jinzhong City, Shanxi Province (37°26′ N, 112°32′ E). Mature flowers and fruits of ‘Jinou 1’ and ‘Nongda 5’ were collected. All materials were stored at −80 °C.

Color parameters (L*, a*, and b* values) of 20 flowers and fruits from each variety were measured using a CR8 colorimeter (3nh, Guangzhou, China). The hue angle H* is a comprehensive color metric calculated by H* = −arctan (b*/a*).

### 4.2. Metabolomic Analysis

The sample was freeze-dried, ground into powder (30 Hz, 1.5 min), and stored at −80 °C until needed. A 50 mg amount of powder was weighed and extracted with 0.5 mL methanol/water/hydrochloric acid (500:500:1, *V*/*V*/*V*). Then, the extract was vortexed for 5 min and ultrasound for 5 min and centrifuged at 12,000× *g* under 4 °C for 3 min. The residue was re-extracted by repeating the above steps again under the same conditions. The supernatants were collected and filtrated through a membrane filter (0.22 μm, Anpel) before LC-MS/MS analysis. Three replicates of each sample were analyzed.

Flavonoids and anthocyanins contents were detected by MetWare (http://www.metware.cn/) based on the AB Sciex QTRAP6500 LC-MS/MS platform was provided by Wuhan Metware Biotechnology Co., Ltd. (Wuhan, China). Principal component analysis (PCA) was performed as described by Yue et al., 2019 [42].

### 4.3. Transcriptome Analysis

Total RNA from flowers and fruits of the ‘Jinou 1’ and ‘Nongda 5’ varieties was extracted using the Trizol reagent as described previously [43]. Based on the sequencing by synthesis, Illumina sequencing was performed at Beijing Biomarker Bioinformatics Technology Co., Ltd. (Beijing, China).

The reads were mapped to the reference *Prunus persica* v2.1 [44] using HISAT2, and the aligned reads were then assembled into transcripts using StringTie [45]. According to the comparison results of HISAT2, the expression levels of all genes in each sample were calculated using the fragments per kilobase of transcript per million mapped reads (FPKM) value as the measurement index of gene expression level.

### 4.4. Differential Expression Analysis and Gene Annotation

DESeq2 v1.46.0 software was used to calculate the expression of 3 biological replicates, and DEGs were analyzed using the criteria |log2FC| > 1 and FDR < 0.05. The identified DEGs were compared using the Non-Redundant Protein Sequence, Nucleotide Sequence, Uniprot, Clusters of Orthologous Groups, Pfam, Gene Ontology, and KEGG databases to obtain gene annotation information [46].

### 4.5. Statistical Analysis

Data were analyzed in Microsoft Excel v. 2023. Pearson’s correlation analysis was also performed using SPSS Statistics 27. Tbtools v2.030 was used to draw figures. Heatmaps illustrating the expression patterns of genes were generated using TBtools [47].

## 5. Conclusions

In conclusion, anthocyanins emerge as the primary determinants of color divergence in floral and fruit tissues between *C. humilis* varieties. Specifically, cyanidin, pelargonidin, paeonidin, and delphinidin were characterized as the dominant pigments driving color polymorphisms in ‘Jinou 1’ and ‘Nongda 5’. Transcriptomic profiling revealed cultivar-specific expression patterns of core anthocyanin biosynthetic genes (*ChCHS1*, *ChDFR*, *ChF3H*, *ChF3’H*), with significantly elevated transcripts in ‘Jinou 1’ relative to ‘Nongda 5’, aligning with its deeper pigmentation phenotype. Critically, integrated metabolome–transcriptome association analyses pinpointed ChMYB9 as a key regulatory hub, exhibiting robust positive correlations with anthocyanin metabolites in both flowers and fruits. These findings advance our mechanistic understanding of floral and fruit color formation in this species and lay a foundation for trait-specific genetic engineering of *C. humilis*.

## Data Availability

The raw data were submitted to the China National GeneBank Database (PRJCA036726 and PRJCA036727).

## Supplementary Materials

The following supporting information can be downloaded at: https://www.mdpi.com/article/10.3390/plants14071103/s1, Figure S1: PCA scores of metabolites in flower (A) and fruit (B). Figure S2: Heatmap of differential metabolites in *C. humilis* flowers; Figure S3: Heatmap of differential metabolites in *C. humilis* fruits; Table S1: A list of metabolites identified in *C. humilis* flowers; Table S2: A list of metabolites identified in *C. humilis* fruits.
