# Supplementary figures and images for: Integrated Transcriptomics and Metabolomics Reveal Key Genes and Metabolic Pathway in Flower and Fruit Color Formation of Cerasus humilis (Bge.) Sok
Source: Plants (Basel). 2025 Apr 2;14(7):1103. doi: 10.3390/plants14071103 (PMC11991490; doi:10.3390/plants14071103)

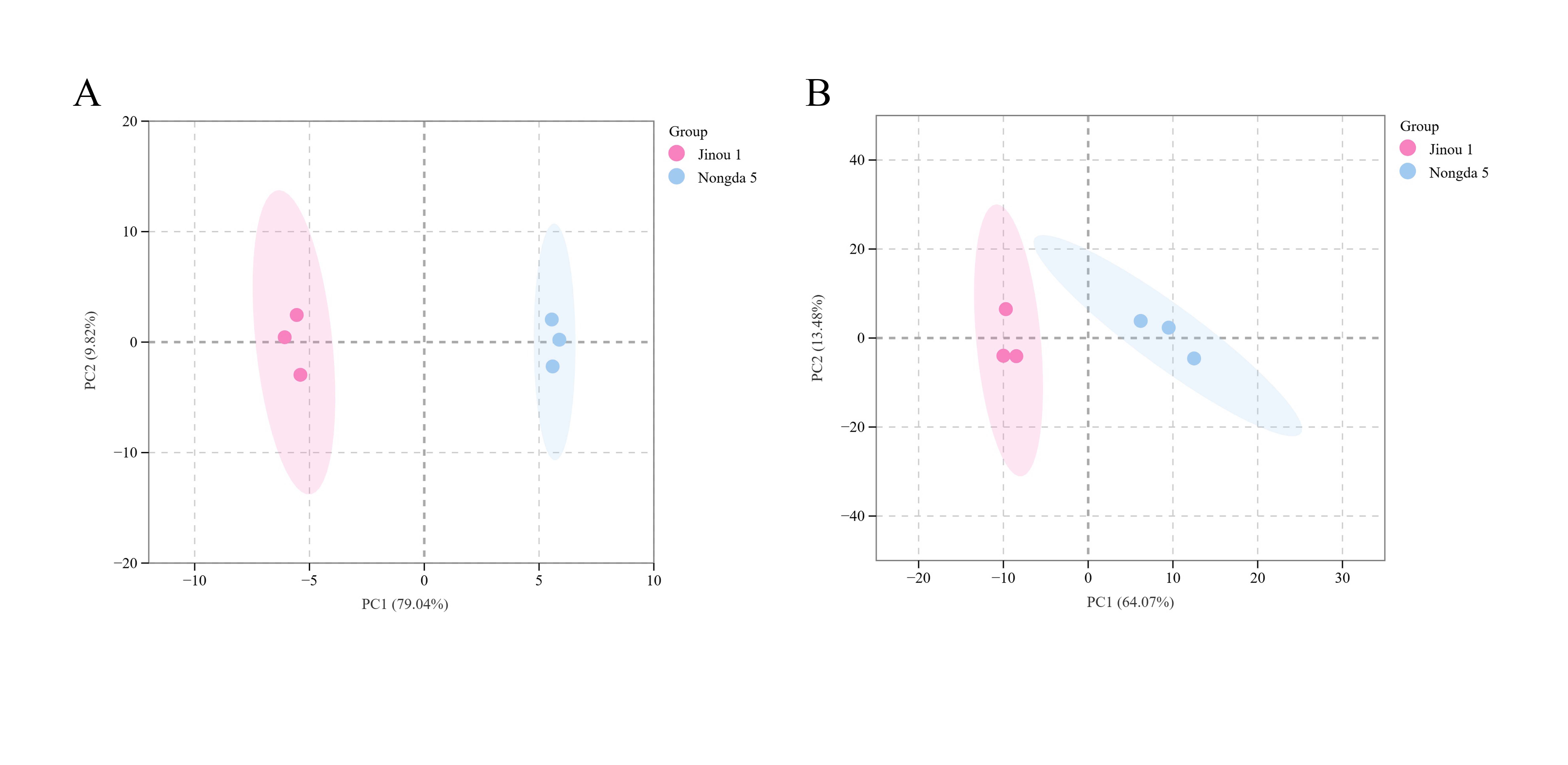

Supplement: Supplementary file 1 [file plants-14-01103-s001.zip › plants-3529747-supplementary-1/Figure S1.jpg]

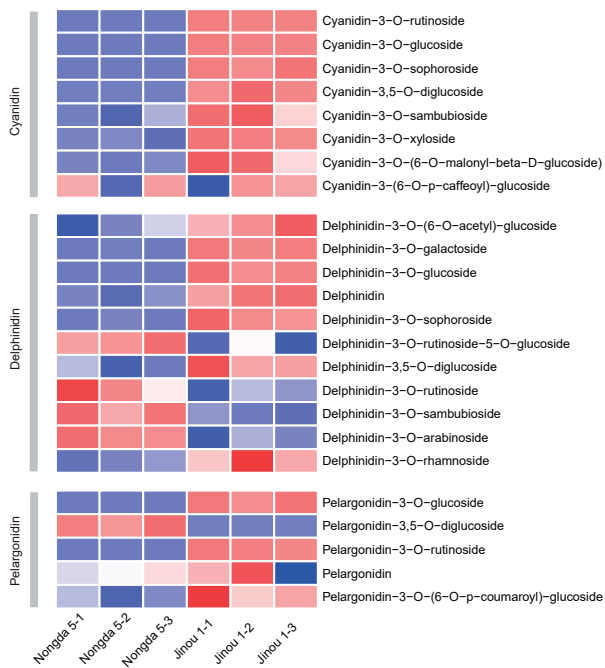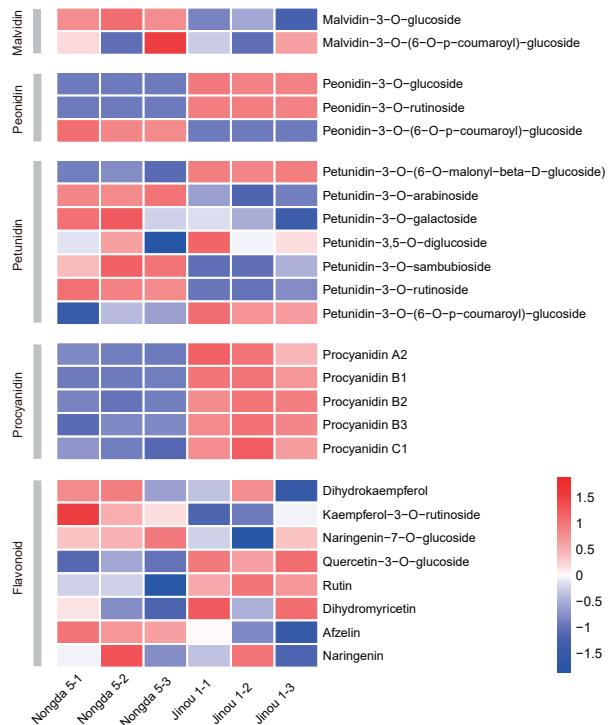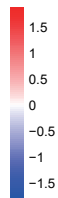

Supplement: Supplementary file 1 [file plants-14-01103-s001.zip › plants-3529747-supplementary-1/Figure.S2.pdf]

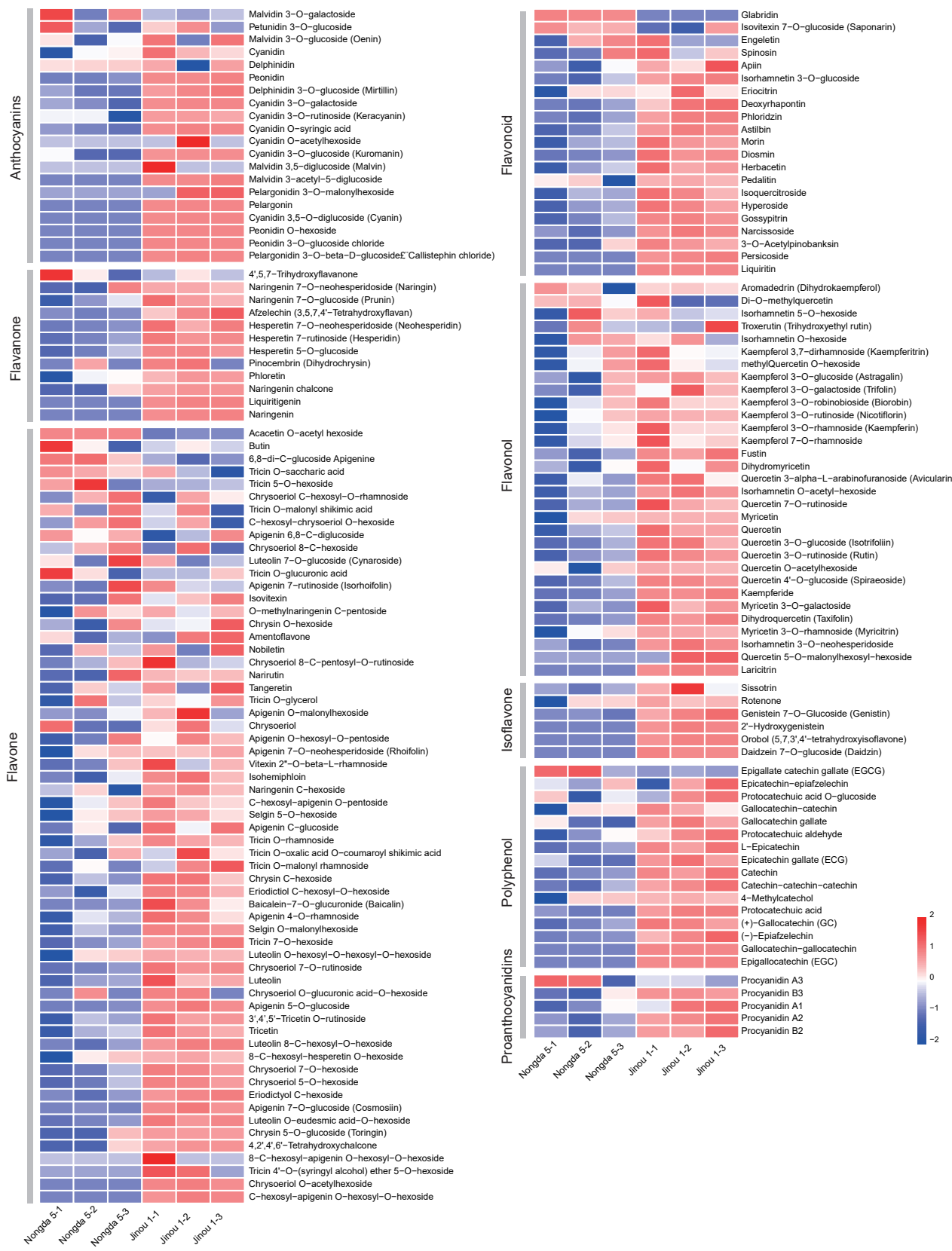

Supplement: Supplementary file 1 [file plants-14-01103-s001.zip › plants-3529747-supplementary-1/Figure.S3.pdf]
